# Alertness and Visual Attention Impact Different Aspects of the Optokinetic Reflex

**DOI:** 10.1167/iovs.62.13.16

**Published:** 2021-10-20

**Authors:** Davide Frattini, Tobias Wibble

**Affiliations:** 1 Department of Clinical Neuroscience, Division of Eye and Vision, Marianne Bernadotte Centrum, St. Erik's Eye Hospital, Karolinska Institutet, Stockholm, Sweden

**Keywords:** visual attention, alertness, ocular torsion, optokinetic nystagmus

## Abstract

**Purpose:**

Assessing visual attention and alertness is of great importance in visual and cognitive neuroscience, providing objective measures valuable for both researchers and clinicians. This study investigates how the optokinetic response differs between levels of visual attention in healthy adults while controlling for alertness.

**Methods:**

Twelve healthy subjects (8 men and 4 women; mean age = 33 ± 9.36) with intact gaze-stability, visual acuity, and binocularity were recruited. Subjects viewed a rotating visual scene provoking torsional optokinetic nystagmus (OKN) while wearing a video eye tracker in a seated head-fixed position. Tasks requiring focused, neutral, and divided visual attention were issued to each subject and the OKN was recorded. Pupil sizes were monitored as a proxy for alertness.

**Results:**

Pupil dilation was increased for both focused and divided visual attention. The number of nystagmus beats was highest for the focused condition and lowest for the divided attentional task. OKN gain was increased during both focused and divided attention. The distribution of nystagmus beats over time showed that only focused attention produced a reliable adaptation of the OKN.

**Conclusions:**

Results consequently indicate that OKN frequency is adaptive to a viewer's level of visual attention, whereas OKN gain is influenced by alertness levels. This pattern offers insight into the neural processes integrating visual input with reflexive motor responses. For example, it contextualizes why attention to visual stimuli can cause dizziness, as the OKN frequency reflects activity of the velocity storage mechanism. Additionally, the OKN could offer a possible venue for differentiating between visual attention and alertness during psychometric testing.

A range of disorders may influence a viewer's attention to visual scenes, including brain trauma, attention-deficit disorders, and a range of neurological and psychiatric conditions.^1^ In healthy individuals, the level of attention can be predictive of accident rates.^2^ Attention is complex, and relies on several key neural structures.^3^^,^^4^ Measuring the activity and outcome of this process offers valuable insight into how individuals distribute their attention, and it can also serve to investigate the impact of any given stimuli. Several techniques have been implemented to objectively evaluate someone's level of attention,^5^^–^^7^ often requiring advanced equipment. Spatial cueing tests use behavioral tasks,^8^ which are widely accessible, but may be influenced by a participant's attributes, such as reflex.

Eye movements offer valuable insight into how visual attention affects human behavior and perception,^3^ and eye-tracking protocols are widely implemented in clinical and scientific studies on visual attention.^9^ With eye-tracking becoming increasingly accessible,^10^ identifying eye-movement parameters influenced by a viewer's attentional level could therefore be of value for developing both clinical and scientific testing protocols. It is evident that various involuntary ocular functions can be correlated to visual attention, including pupillary responses^11^^–^^13^ and microsaccades.^14^^,^^15^ The optokinetic reflex (OKR), causing optokinetic nystagmus (OKN), aims to clamp a moving visual scene onto the retina in order to avoid retinal slip.^16^ An OKR relies on basic subcortical structures,^17^^,^^18^ although in mammals it also receives substantial cortical influences.^19^ Depending on a human viewer's state of mind they may subconsciously modify the nature of the OKR. For instance, a decrease in attentional focus will decrease the OKN,^20^ whereas a heightened attention to visual motion has been shown to increase the OKN in humans.^21^^–^^23^ There is currently no study comparing OKN responses between focused and divided attentional levels within a human observer.

The OKN benefits from offering an objective indication of a viewer's level of visual attention, without being influenced by voluntary commands. Torsional OKN (tOKN) is seen in response to rotating visual scenes,^24^ and although tOKN has been the least investigated type of OKN, bedside assessment tools are emerging.^25^ Unlike horizontal and vertical eye movements, there is no meaningful somatic control of ocular torsion, minimizing the risk of confounding voluntary gaze shifts. Therefore, tOKN has been shown to reflect the visual clutter of a moving scene, increasing in gain,^26^ and also correlate with a viewer's stress response to visual motion, as reflected in the pupil size.^27^ Heightened alertness is also known to increase the pupil size, reflecting increased autonomic signaling.^28^^,^^29^ Whereas alertness can be categorized as a subtype of attention, they operate through separate neurophysiological networks.^30^ Implementing tOKN to study the effects of visual attention and alertness may consequently offer new insights into visual motion processing.

This study aims to investigate how the OKN may be used to indicate a person's level of attention to visual motion. Eye tracking will be used to quantify the tOKN to tasks requiring focused and divided levels of visual attention. Pupil size will serve as an indication of the level of alertness. We hypothesize that focused visual attention will enhance the OKN, whereas divided attention will inhibit it. These responses may indicate how attention influences the neural processing integrating visual motion and its impact on visually guided behaviors.

## Methods

### Subjects

The study was carried out with 12 healthy participants (4 women and 8 men, mean age = 33 years, SD 9.29), recruited from university staff and colleagues due to convenience and coronavirus disease (COVID) restrictions. This sample size was chosen as it is similar to those of previous studies in the field.^22^^,^^31^ All subjects exhibited normal corrected visual acuity (VA; ≥1.0), stereoscopic vision (TNO ≤60 inches), as well as normal vestibular ocular reflex and eye motility, as tested by the head impulse-test and ocular motility test. Exclusion criteria included any history of attention deficit disorders or problems relating to concentration. The research protocol adhered to the Declaration of Helsinki. All participants received written and oral descriptions regarding the nature of the study and provided written consent at the time of recruitment. The ethical permit was approved by the Regional Ethics Committee of Stockholm (EPN 2018-1768-31-1).

### Procedure

All participants were exposed to three sets of optokinetic stimulations, requiring different levels of visual attention. These were referred to as focused, neutral, and divided attentional levels (see below). In order to control for alertness, focused and divided attention trials were designed to demand significant sensory alertness relative to the neutral condition.

The order of attentional tasks was presented according to stratified randomization in a balanced manner and participants were informed of the nature of the tasks prior to initiating the trials. The optokinetic responses were evaluated using a video eye-tracker, recording torsional eye movements for analysis. Recordings were carried out in a dimly illuminated room at a fixed luminosity so as not to affect alertness levels or change the visual contrast between trials. Subjects’ heads were positioned on a height-regulated chinrest 60 cm away from the screen (Sharp LCD 55 inch, 50 Hz; Sharp Electronics, Hamburg, Germany), thus ensuring a correct alignment between the primary gaze position and the center of the screen; the latter featured a fixation point where subjects were asked to stabilize their gaze. The screen covered a visual angle of 90 degrees horizontally and 59 degrees vertically. Any eye movement deviating from the primary eye position was identified in real-time so as to minimize the risk of secondary ocular torsion caused by oblique eye movements. The tOKN frequency and gain were recorded using at 100 Hz head-mounted Chronos Eye Tracker (CET; Chronos Inc., Berlin). The data was retrieved and analyzed according to well-established and previously published procedures.^26^^,^^27^^,^^32^

### Optokinetic Stimulation

The optokinetic stimulation consisted of a high-resolution image of 26 black lines, 1.5 cm wide, and 16 cm long, against a white background. Visual angle of the lines was 15.18 degrees and they stood at an angle of 45 degrees, rotating 1440 degrees counter-clockwise with a velocity of 72 degrees/s around a central fixation point, which was 1 cm in diameter and at a visual angle of 0.96 degrees. Each trial started with the stimulus being presented at rest for 10 seconds, followed by 20 seconds of optokinetic stimulation, before ending with 5 seconds of static presentation. This allowed the visual scene to rotate four full circles, starting and ending in the same position and orientation. The optokinetic stimulation is represented in Supplementary Figure S1.

### Attention Levels and Tasks

The protocol included three different levels of attentional engagement depending on the visual stimulation: focused, neutral, and divided. In the condition with focused attentional engagement on the visual stimulation, participants were instructed to verbally report each rotation of the optokinetic stimulation while maintaining gaze fixation on the fixation point; this was performed simultaneously with the stimulation. The number of correct rotations reported represented the attentional engagement score, with four points accounting for a perfect performance.

In the condition with the neutral level of attentional engagement on the visual stimulation, participants were tasked with simply viewing the visual stimulation while maintaining their gaze on the central fixation point. No task was given and subjects were instructed to relax and simply sit through the protocol. The condition with divided attentional engagement on the visual stimulation featured a distracting task being presented to the participants simultaneously to the visual stimulation. To ensure a shift of the attentional focus away from visual to auditory cues, the Paced Auditory Serial Addition Test (PASAT) with a 2-second interval between stimuli^33^ was submitted to the participants. The PASAT consists of a sequence of digits presented through a speaker, with each digit separated by a fixed interval. The subjects were asked to add consecutive digits in response to the numbers called out and immediately report the sum verbally. The PASAT started 5 seconds before the active optokinetic rotation and ended 5 seconds after it had stopped.

### Statistical Analysis

Attentional tasks were compared using repeated measures ANOVA with α of 0.05 for torsional slow-phase velocities, number of nystagmus beats and peak torsional amplitudes. Mauchly's sphericity test was implemented for each variable. Post hoc analyses of the ANOVA results were performed with Holm corrections for comparing families of three. Statistical analyses were performed using JASP (version 0.9.2; JASP Team 2019) or SPSS (SPSS Statistics 25 for Windows; IBM, Armonk, NY, USA). Significance levels in figures are given as ns = not significant, * = *P* ≤ 0.05, and ** = *P* ≤ 0.01.

A repeated measures ANOVA was performed on the pupil size data. Post hoc comparisons with Holm corrections were used to compare pupil sizes between focused and divided attentional tasks to those during neutral viewing. In addition, correlation analyses were performed to assess the relationship between pupil size and the torsional nystagmus parameters. In addition, the OKR is known to adapt over time.^34^ In order to examine the effects of attention on this adaptation, correlation analyses were performed between the onset of nystagmus beats and their respective velocities throughout the active stimulation phase.

## Results

### Establishing Alertness Levels

Pupillometry revealed that pupil sizes during both the focused (32.65 ± 2.15, *P* = 0.017) and divided (35.03 ± 3.50, *P* = 0.005) attentional tasks caused increased pupil sizes relative to the neutral condition (30.85 ± 3.00; F(2, 22) = 17.851, *P* < 0.001). This indicated that both conditions heightened subjects’ level of alertness. Correlation analyses revealed no significant relationship between pupil size, torsional velocities, or number of nystagmus beats. This analysis was performed both within each viewing condition, as well as on pooled data. There was consequently no evidence indicating that the nystagmus response was influenced by alertness.

### Attentional Influences on the Optokinetic Reflex

**Figure 1.**
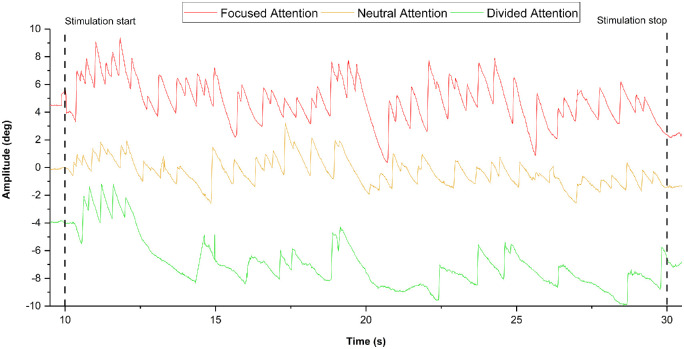
**Representative traces of the optokinetic eye movement responses during different states of visual attention**. The torsional response to focused (red), neutral (orange), and divided (green) attentional levels for one subject. These results illustrate how increased visual attention upregulates the optokinetic nystagmus. Starting amplitudes have been adjusted to allow for simultaneous presentation of all traces, and blinks have been replaced by bridging lines across data points.

**Figure 2.**
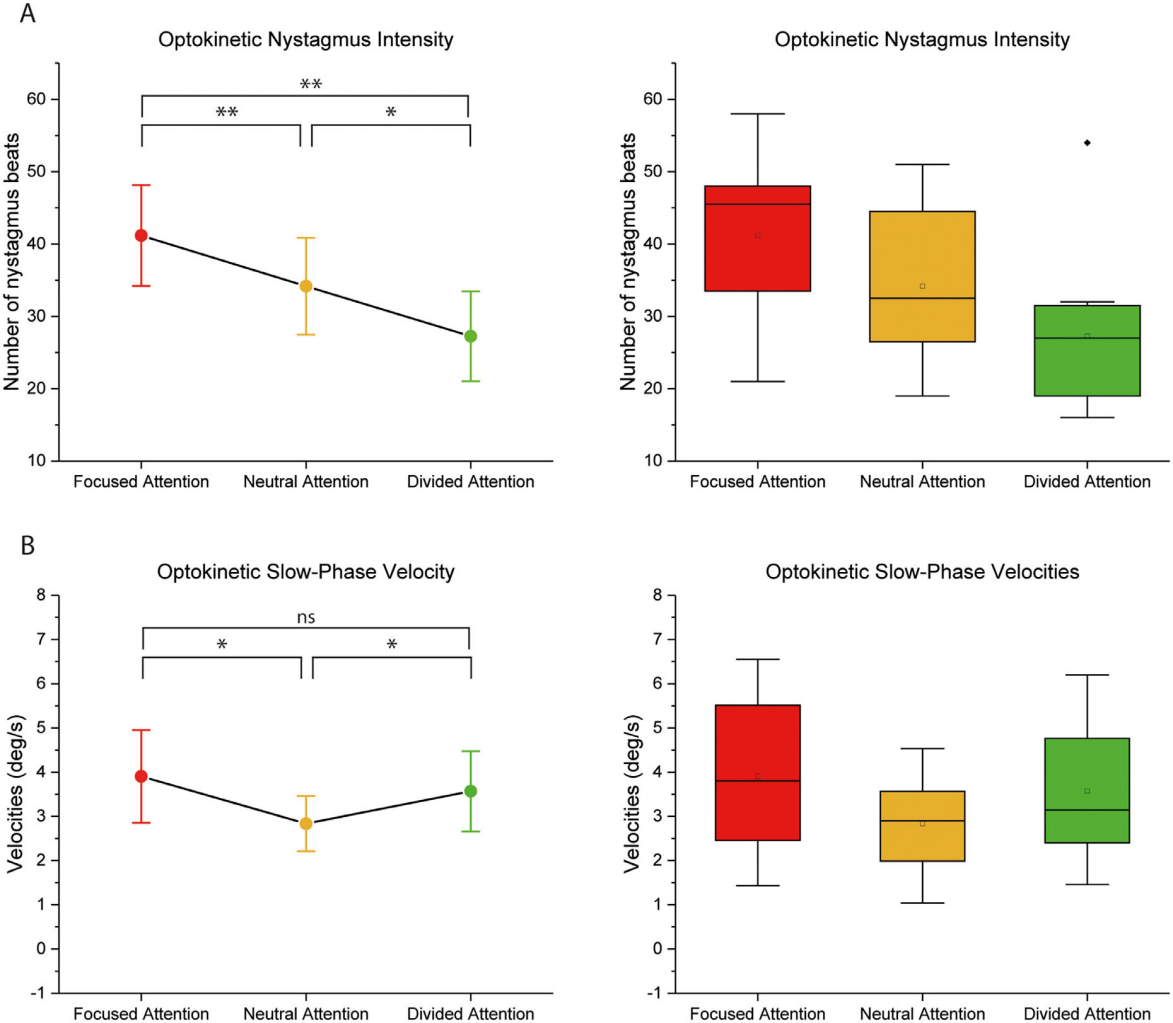
**Optokinetic eye movement responses during different states of visual attention**. (**A**) An interval plot outlining the mean and SD of the number of nystagmus beats for each attentional level, highlighting the correlative relationship. To the right is a descriptive box-plot reflecting the data supporting plot A, showing mean (square), SD (colored box), median (horizontal line), and 95% confidence interval (error bars). (**B**) An interval plot outlining the mean and SD of the number of nystagmus beats for each attentional level, showing increased responses during both focused and divided attentional tasks compared to neutral viewing. To the right is a descriptive box-plot reflecting the data supporting plot B, showing mean (square), SD (colored box), mean (horizontal line), and 95% confidence interval (error bars).

Representative traces of the OKR for all attentional levels are illustrated in Figure 1. There was a significant effect of attentional task on torsional velocities (F(2, 22) = 7.144, *P* = 0.006). As illustrated in Figure 2, both focused (*P* = 0.026) and divided (*P* = 0.049) attentional conditions yielded significantly greater velocities compared to the neutral condition. Attention had no significant effect on the peak slow-phase amplitude, although sphericity could not be assumed for this variable. Quick-phase amplitudes were similarly unaffected. These findings reflect that both focused and divided attentional focus upregulates the OKR velocity without altering the amplitude. This means that the increased OKN gain was likely due to increased levels of alertness.

The number of nystagmus beats, reflecting the activity of the velocity storage, was greatly influenced by the level of attentional focus. As illustrated in Figure 2, the number of beats was highest for the focused task and lowest for divided condition, with the results from neutral trials aligning in between (F(2, 22) = 12.353, *P* < 0.001). Post hoc analyses show significant differences among all three attentional focus conditions; focused to neutral (*P* = 0.010), focused to divided (*P* = 0.004), and divided to neutral (*P* = 0.050).

Altogether, this indicates that greater attentional focus is associated with increased activity in the velocity storage mechanism. This means that the more attention the subject paid to the visual stimulus, the greater its influence on the viewer's neural integration of motion.

### Temporal Distribution of the Optokinetic Responses

**Figure 3.**
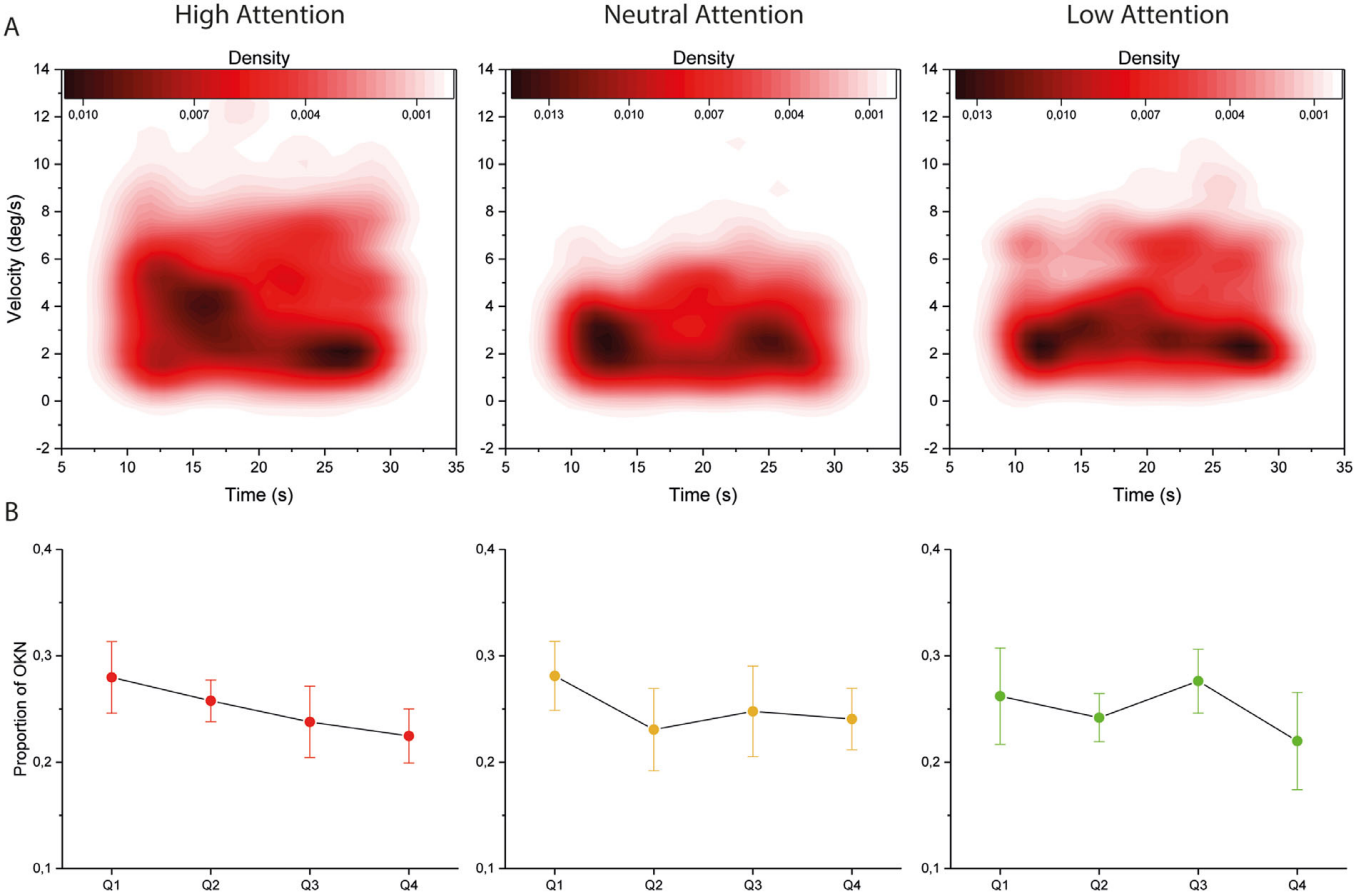
**The distribution of slow-phase velocities over time.** (**A**) The Kernel density plots signify distribution density of OKN slow-phases across the stimulation period, with darker regions corresponding to increased number of data points for the eye movement velocity at that point in time. (**B**) The interval plots represent the proportion of OKN beats for each quarter (Q1–Q4) throughout the stimulation period for each attentional level, presented on a scale of 0 to 1, also showing the standard deviation. The focused task caused a steady decline in relative OKN distribution, whereas the neutral task evens out after an initial cluster of beats. The divided task yielded clusters in the first and third quarter, though with a decreasing trend toward the fourth quarter. A similar trend can be seen for the neutral condition.

In order to assess if attention levels had any significant effect on OKN adaptation over time, a Pearson's correlation was performed between the starting time of each slow-phase and its corresponding velocity across all subjects. No significant correlation could be found for any attentional level. However, the general distribution of the slow phases was noticeably affected by attentional focus (Fig. 3A). To further investigate how the attentional level influences the OKN over time, the number of OKN beats were calculated for each quarter of the stimulation period. A repeated measures ANOVA revealed a significant interaction effect between the attentional task and the quarterly distribution of OKN beats at the group level (F(2, 6) = 4.298, *P* = 0.050). To illustrate this relationship, each quarter was plotted as a percentage of the total number of OKN beats for each attentional level (Fig. 3B). These results indicate that OKN adaptation over time differs based on the viewer's attention to visual motion.

### Individual Considerations in the Attentional Tasks

As both focused and divided attentional tasks rely on individual strategies, each subject was asked about their method of approaching both tasks. This was carried out after all measurements had been collected, so as to not influence the subject. The divided attentional task, featuring the PASAT, was not associated with any specific strategy in general, with all subjects being naïve to the procedure apart from the brief introduction outlined in the methods section. To assess any relationship between success rates in the divided attentional task and the OKN, Pearson's correlation coefficients were retrieved between the PASAT scores and eye movement parameters, revealing no significant correlation.

All subjects reported the same strategy for resolving the focused attention task. This was done by maintaining focus on the line closest to the gaze-fixation point, keeping a mental note of when it passed by its estimated starting position. All subject achieved a perfect score for estimating the number of rotations. For this reason, no further statistical evaluation was warranted.

## Discussion

This study outlines the effects of visual attention on the optokinetic response, showing how the velocity storage mechanism is deeply linked to the level of attention an individual commits to a moving visual scene. As reflected by the number of nystagmus beats, the divided visual attention is associated with decreased motion processing, whereas an increase is seen for tasks demanding focused visual focus. The fact that OKN gain and distribution density was increased to both divided and focused attentional levels, both requiring high alertness, highlights the complexity of visual sensorimotor integration.

### The Influence of Alertness

Pupil data indicated that both focused and divided attentional tasks heightened viewers’ alertness levels compared to the neutral condition. The study design consequently allowed us to control for alertness. The fact that no relationship was found between pupil size and nystagmus responses is in contrast to previous findings having shown that pupil size was moderately correlated to torsional slow-phase velocities.^27^ This may be due to the fact that this study implemented a much smaller screen which subjects viewed while seated, meaning that they did not have to perform any postural shifts to counterbalance the vection produced by a bigger scene movement. Ultimately, these results indicate that pupil sizes could not explain the changes in nystagmus parameters. Differences in the eye movement response between focused and divided attentional tasks may therefore be attributed the attentional level, as seen in the analysis of OKN frequency. Similarities between the two tasks may instead be due to the level of alertness, here illustrated in the OKN gain.

### The Effects of Visual Attention

In order to contextualize these results, it is important to identify that optokinetic nystagmus is a gaze-stabilizing eye movement which reflects a need to maintain visual integrity during motion. The two primary mechanisms underlying gaze stability are the vestibulo-ocular reflex (VOR) and the OKR, which are thought to have evolved in parallel.^35^ These are interlinked to allow a multisensory estimation of movement, and visual motion is conveyed to the vestibular nuclei through the optic nerves,^36^ and it is well-known that retinal flow modifies the vestibular velocity storage mechanism (VSM).^37^ This system is geared to allowing motion perception during sustained vestibular stimulations.^38^ As seen in this study, the impact on the VSM was readily modified by the level of visual attention. The fact that focused visual attention correlates with greater VSM activity fits well within the framework of patients suffering from non-vestibular vertigo due to increased visual motion sensitivity.^39^ Indeed, several patient groups presenting with visually induced vertigo have been shown to exhibit increased nystagmus activity to optokinetic stimuli,^40^^,^^41^ and diverting attention can alleviate the effects of visual motion hypersensitivity.^42^ It is also noteworthy that the clear adaptation of the OKN over time was only observed during focused viewing, with neutral and divided attentional tasks being associated with greater fluctuations in OKN frequencies. This finding further supports the claim that visual attention, more so than alertness, influences the VSM. In light of this, the OKN may prove a valuable proxy for assessing visual attention, and allow personalized rehabilitation through following the recorded data over time.

However, as previously addressed, attention and alertness are separate neural entities, although conceptually related.^30^ Our results further support this notion, as reflected by torsional velocities not adopting the same pattern as the number of OKN, with both focused and divided levels of visual attention increasing the OKN gain. These results indicate that alertness may have influenced the tOKN gain. The PASAT protocol inducing divided visual attention necessitates a broad activation of cortical structures, including the left prefrontal cortex and left parietal lobe and visual associative areas.^43^ As these structures are involved in producing eye movements, one may hypothesize that the voluntary suppression of the OKN could have been downregulated during the stimulation period, and PASAT tasks have indeed been shown to downregulate the efficacy of gaze shifts.^44^ Studies in rabbits have shown that alertness, caused by vibration, shortens the OKN time constant while also increasing the frequency of updated OKN accelerations.^45^ Comparing the eye movement results to the corresponding pupil size data, this study indicates that both focused visual attention and heightened alertness will result in increased OKN velocities, supporting the notion that this relationship is also true for humans.

The methodological approach in this study did produce some limitations in how the data may be interpreted. Through continuously providing PASAT answers, the subjects were confirmed to be engaged with the task, although it is difficult to assess the exact level of attention within individual subjects. PASAT proficiency does not depend on a certain level of neural activation, and so it is not surprising that no correlation could be seen between the number of correct answers given and the OKN. In addition to the number and gain of the OKR, this study investigated the effects on peak amplitudes, both for slow and quick phases. The main aim of calculating peak amplitudes was to assess whether the attentional level would affect the integration of stimulation amplitudes into the OKR (i.e. if increased attentional levels would correspond to the movement being treated as covering a greater distance). There was, however, no significant effect on this outcome based on the attentional task. Peak slow-phase amplitudes also produced a high degree of variance. It should be noted that the OKN gain was calculated from each slow phase for every trial, whereas the peak amplitudes were retrieved twice per trial, one positive and one negative. This meant that the number of traces available for statistical analysis were much lower for peak amplitudes. The comparative lack of power naturally limits the contextualization of how OKN peak amplitudes are affected by the attentional tasks.

In conclusion, this study is the first in implementing a range of perceptual tasks to show that visual attention and general alertness influences separate aspects of the optokinetic reflex. The number of OKN beats was highest during focused levels of visual attention and lowest during the divided attention task, reflecting greater involvement of the velocity storage mechanism in integrating visual motion. In addition, OKN gain increased during both divided and focused attention levels. This was also true for the pupil size, which may reflect that the increased gain was due to a heightened alertness. In addition, the temporal distribution of the OKN suggests that focused visual attention stimulates adaptation of the visual motion processing system to a greater extent than heightened alertness. These factors indicate that features of the OKN are influenced independently by attentional and alertness levels. Altogether, the OKN reflects different aspects of cortical and subcortical sensorimotor processes important for human visual perception, and reflects primordial attentional cues relating to visually guided behaviors. Moving forward, these findings may allow for objective correlates to attention during psychometric testing, as well as personalized clinical protocols when treating patients with attentional disorders.

## Supplementary Material

Supplement 1
