# Supplementary material for: Alertness and Visual Attention Impact Different Aspects of the Optokinetic Reflex
Source: Invest Ophthalmol Vis Sci. 2021 Oct 20;62(13):16. doi: 10.1167/iovs.62.13.16 (PMC8543398; doi:10.1167/iovs.62.13.16)
Supplement: Supplement 1 [file iovs-62-13-16_s001.pdf]

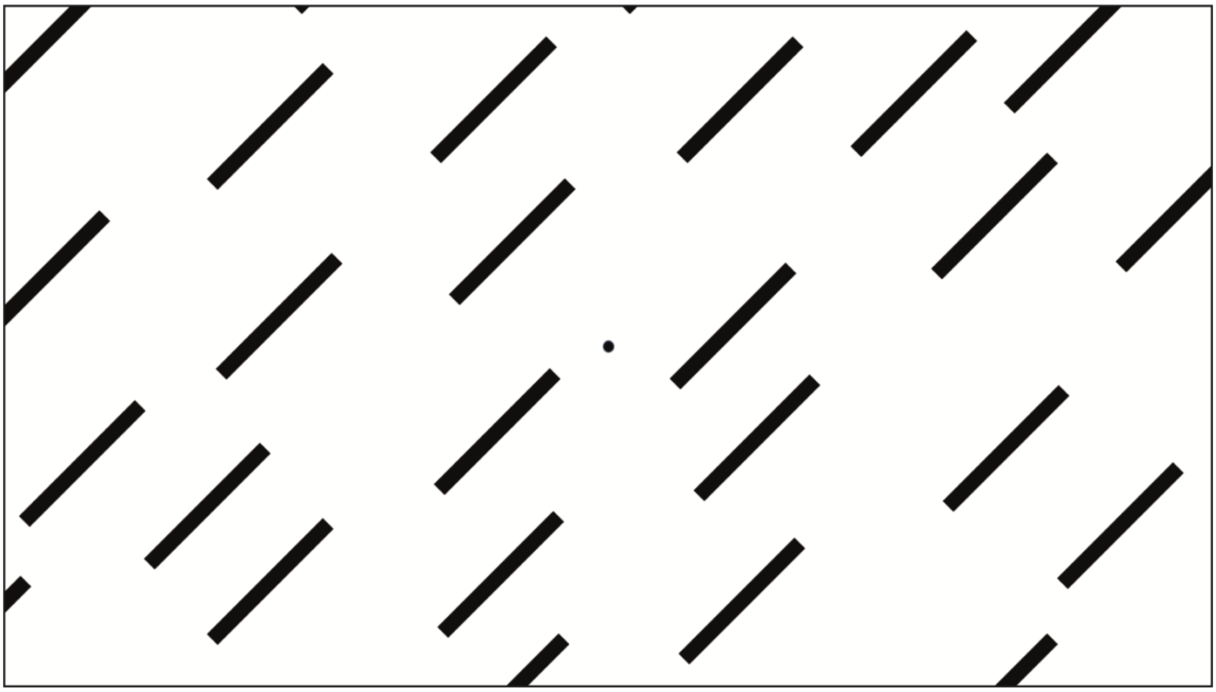

Supplementary figure 1. The optokinetic stimulation. The visual scene consisted of 26 black lines (1.5cm wide and 16cm long, visual angle 15.18 deg.). The lines were centred on a fixation point with a diameter of 1cm, visual angle 0.96 deg., and at a 45 deg. inclination in relation to gravitational upright. The active stimulation involved the entire scene rotating 1440 degrees counter-clockwise with a velocity of 72 deg./s for 20 seconds, accounting for 4 full rotations.
